# Reconciling validity and challenges of patient comfort and understanding: Guidelines to patient‐oriented questionnaires

**DOI:** 10.1111/hex.13373

**Published:** 2021-10-20

**Authors:** Catherine Hudon, Alya Danish, Mireille Lambert, Dana Howse, Monique Cassidy, Olivier Dumont‐Samson, Judy Porter, Donna Rubenstein, Véronique Sabourin, Shelley Doucet, Vivian R. Ramsden, Mathieu Bisson, Charlotte Schwarz, Maud‐Christine Chouinard

**Affiliations:** ^1^ Département de médecine de famille et de médecine d'urgence Université de Sherbrooke Sherbrooke Canada; ^2^ Centre de recherche du centre hospitalier universitaire de Sherbrooke Sherbrooke Canada; ^3^ Centre intégré universitaire de santé et de services sociaux du Saguenay‐Lac‐Saint‐Jean Chicoutimi Canada; ^4^ Primary Healthcare Research Unit Memorial University St. John's Canada; ^5^ Department of Interdisciplinary Studies University of New Brunswick Saint John Canada; ^6^ Patient Partner Canada; ^7^ Department of Nursing and Health Sciences University of New Brunswick Saint John Canada; ^8^ Department of Academic Family Medicine University of Saskatchewan Saskatoon, Saskatchewan Canada; ^9^ Faculté des sciences infirmières Université de Montréal Montreal Canada

**Keywords:** guidelines, patient engagement, patient‐reported outcome measure, questionnaire

## Abstract

**Background:**

Patient‐reported outcome measures (PROMs) are widely recognized as important tools for achieving a patient‐centred approach in health research. While PROMs are subject to several stages of validation during development, even questionnaires with robust psychometric properties may challenge patient comfort and understanding.

**Aim:**

Building on the experience of patient engagement in the PriCARE research programme, this paper outlines the team's response to concerns raised by patient partners regarding the administration of the questionnaire.

**Methods:**

Based on a participatory action research design and the patient engagement framework in the Strategy for Patient‐Oriented Research of the Canadian Institutes of Health Research, PriCARE team members worked together to discuss concerns, review the questionnaires and come up with solutions. Data were collected through participant observation of team meetings.

**Results:**

This paper demonstrates how patient partners were engaged in PriCARE and integrated into the programme's governance structure, focusing on the challenges that they raised regarding the questionnaires and how these were addressed by PriCARE team members in a six‐step approach: (1) Recognizing patient partner concerns, discussing concerns and reframing the challenges; (2) Detailing and sharing evidence of the validity of the questionnaires; (3) Evaluating potential solutions; (4) Searching the literature for guidelines; (5) Creating guidelines; and (6) Sharing and refining guidelines.

**Conclusion:**

This six‐step approach demonstrates how research teams can integrate patient partners as equal members, develop meaningful collaboration through recognition of individual experiences and expertise and ensure that the patient perspective is taken into consideration in research and healthcare innovation.

**Patient or Public Contribution:**

All patient partners from the PriCARE programme were actively involved in the six‐step approach. They were also involved in the preparation of the manuscript.

## 1 INTRODUCTION

It is now widely recognized that involving patients in healthcare research helps to capture what is most important to them. Patient‐reported outcome measures (PROMs) are one of the primary tools in the philosophy of a patient‐centred approach to assess care. They highlight what really matters in the patient's life and can measure the impact of an intervention from the patient's perspective.^1^ As stated by international guidance, questionnaires of PROMs are subject to several stages of validation, such as identifying the conceptual framework of the measure and assessing validity and fidelity.^2^, ^3^ During the process of validation, the FDA^2^ and the International Society for Quality of Life Research^3^ recommend involving patients in the development of the PROM, for example, by interviewing patients to establish or confirm its face validity. Still, there remains a large variation in the levels and methods of patient engagement.^4^, ^5^, ^6^, ^7^, ^8^ Despite recommendation from the scientific community to avoid the reformulation of questions after a PROM has been validated,^9^, ^10^ even questionnaires with robust psychometric properties may pose challenges for vulnerable populations. These challenges can include lack of clarity, degree of comfort answering certain questions or the applicability of a generalized questionnaire to the particular, complex situation of the individual. The language used, or the way in which the questions are presented, may not always be clear to patients,^11^, ^12^ and the collection of demographic data may make patients uncomfortable.^13^

Questionnaires may be challenging for certain vulnerable groups, such as people with cognitive impairments, multimorbidity or low literacy levels. For people with cognitive impairments, quantitative evaluation, comparison questions, abstract concepts and generalized statements can be challenging.^14^ The unique situation of patients with multiple chronic conditions is not always taken into account in the way in which questions are formulated.^6^ Individuals belonging to some vulnerable populations may have difficulty in understanding rating scales and mutually exclusive response choices or may find them too complex.^15^, ^16^, ^17^, ^18^ Rigid adherence when administering questionnaires to older people may inhibit the interviewer from interacting and engaging meaningfully with the respondent or responding to cues indicating distress or grief.^17^

To overcome some of these challenges, Boynton et al.^16^ propose a guide to questionnaire research for ‘reaching beyond the white middle classes’. For example, they suggest placing demographic questions at the end of the questionnaire to minimize the threat for participants. Further, they emphasize the importance of explaining clearly why the information is required and how it will be protected. They also suggest working closely with community representatives and conclude that the quality of social interactions during questionnaire administration undoubtedly influences responses.

The inclusion of patients as genuine partners on research teams requires researchers to be open and responsive to their feedback. Building on the experience of the PriCARE team, this paper proposes (1) steps to address the challenges of patient comfort and understanding of the questionnaires and to reach consensus as a team, and (2) patient‐oriented guidelines for administering the questionnaires.

## 2 MATERIALS AND METHODS

### 2.1 The PriCARE research programme

The PriCARE research programme^19^, ^20^ was funded by the Canadian Institute of Health Research in the Strategy for Patient‐Oriented Research (SPOR)—Primary and Integrated Health Care Innovation (PIHCI) Network programmatic grants. The programme's overarching goals are to (1) generate findings on the implementation of case management (CM) in primary care for individuals with chronic conditions and complex healthcare needs who frequently use healthcare services and (2) implement an evidence‐based intervention to improve care experience and outcomes and to guide policy decision‐making.

The PriCARE research programme is a multiple‐case embedded mixed‐methods study conducted in five participating Canadian provinces: Newfoundland and Labrador, Nova Scotia, New Brunswick, Quebec and Saskatchewan. One or two primary care clinics were recruited in each province to implement and evaluate the CM intervention among frequent users of healthcare services with chronic diseases and complex care needs. The intervention is detailed elsewhere.^19^ To fulfil the inclusion criteria for the programme, participants must be over 18 years of age, living with at least one chronic condition (including mental health), have complex healthcare needs, frequent users of healthcare services according to professional judgement and likely to benefit from the intervention. Patients with loss of autonomy, living in a long‐term care residence, having a prognosis of less than one year or unable to consent were excluded.

In the PriCARE research programme, we conducted a participatory action research project in which both academic and nonacademic researchers collaborated closely.^21^ This design focuses on enabling action through a reflective process, in which stakeholders participate in the data collection and analysis, and identify areas of action in collaboration with academic researchers.^22^ Participatory research aims to improve the participation of the population for whom the research results are intended, to mitigate power imbalance and to recognize and promote the value of lived experience. In PriCARE programme research, researchers (principal and coinvestigators, research assistants and a postdoctorate fellow) work closely with patient partners.

In line with the SPOR of the Canadian Institutes of Health Research, PriCARE programme research actively engages patient partners as equal team members in all aspects of the research process.^23^, ^24^ A community of patient partners was formed, including six patient partners, one or two per province, who worked closely with the research team. These patient partners were recruited based on their personal experience of having complex healthcare needs or as a family member of someone with complex care needs. The patient partners were integrated into the programme's governance structure as members of the steering committee to ensure their engagement in all research phases and that their priorities remained a focus. Patient partners were involved in all stages of the PriCARE research programme: formulation of the research objectives, review of data collection tools, training of the case managers, recruitment of participants, programme monitoring, data analysis and interpretation and drafting of publications. One of the roles of patient partners was to represent the voice of patients with complex care needs. The terms of reference agreed on by all PriCARE team members specified the terms and conditions of patient engagement in the research programme, that is, purpose, role, activities, working procedures, compensation and confidentiality.

### 2.2 The patient questionnaires

The PriCARE research programme uses a mixed‐method data collection approach, including patient questionnaires. Based on a literature review, instruments were carefully selected to measure several variables, such as health literacy,^25^, ^26^ multimorbidity,^27^, ^28^ care integration,^29^, ^30^ self‐management,^31^, ^32^ quality of life^33^, ^34^ and psychological distress.^35^, ^36^ Inclusion criteria were as follows: ability to be self‐administered, good psychometric properties, availability in English and French and low number of items. All instruments selected were short (≤21 items), reliable and valid in both languages. See the Supporting Information Appendix S1 for more details.

### 2.3 The challenges with the questionnaires

During a meeting with a research assistant, patient partners reviewed the patient questionnaires, which were comprised of several prevalidated instruments (Supporting Information Appendix S1), and expressed reservations regarding the general administration of the questionnaires, as well as with specific questions. First, patient partners indicated that going through the questionnaires was an uncomfortable experience. They emphasized the personal nature of some of the questions, noting that certain questions could be triggering as a result of past negative healthcare experiences, and being asked to reflect on difficult topics. These topics included their ability to care for themselves, their experiences with physical or psychological distress as a result of multiple chronic illnesses and mental health challenges. Patient partners emphasized the importance of establishing a relationship of trust between study representatives and participants by starting the administration process slowly, reviewing the purpose of the questionnaire, and easing into more difficult questions to help alleviate discomfort.

Second, patient partners desired clarification on why participants were being asked such questions, and whether, or how, their responses would be beneficial. They felt that further justification was needed for particularly sensitive or invasive questions for participants to feel comfortable sharing their responses.

Third, patient partners found some of the questions confusing or found the wording difficult to understand. They felt that there were several ways to interpret these questions, and that participants would need assistance, as the response choices did not always reflect the type of answer that participants would have thought to provide.

Fourth, patient partners felt that some of the questions were phrased in a way that presumed that the respondent had a single health condition and did not adequately reflect the realities of living with multiple chronic conditions. For example, when considering a question concerning health literacy, patient partners noted that some of the respondents' illnesses may be well managed by the participant, while others may be more challenging and less understood. Thus, patient partner participants would find it difficult to answer questions that did not account for multiple, complex needs. Patient partners expressed interest in adding, removing or revising some of the questionnaire items and felt that some of them seemed outdated. It is important to note that, in general, many patients suffer from multiple chronic conditions, often alongside mental health conditions and/or social vulnerabilities,^37^, ^38^ which may interfere with their ability to complete a questionnaire.^16^

These concerns were discussed during a PriCARE team meeting and the team members decided to find solutions to address these concerns.

### 2.4 Data collection and analysis

Participant observation of eight virtual PriCARE team meetings was used to collect data on the concerns raised by patient partners regarding questionnaire administration and the team's response. The meetings observed were 1 h community of patient partner and steering committees scheduled between 27 January and 22 June, a period where the team members recognized and addressed these concerns. The team members included six patient partners, eight academic researchers, one postdoctoral researcher and five research assistants. Data were collected through the minutes from meeting and notes taken by one of the authors (A. D.). The data were analysed by two authors (A. D. and C. H.) using thematic analysis^39^ to capture the challenges raised by the patient partners regarding the questionnaires, as well as the steps taken by PriCARE team members to address these challenges. Various techniques were used to ensure the trustworthiness (validity and reliability).^40^ Credibility of the results was ensured through persistent observations, prolonged engagement of the authors and member checking. Transferability of the findings was achieved through thick description of the context of the study. Dependability of the results was ensured by a description of the data collection and analysis. Confirmability of the findings was ensured through researchers' triangulation and team validation.

This study was approved by the ethic review boards of the Centre intégré universitaire de santé et services sociaux de l'Estrie‐CHU (Quebec, Canada). All participants completed and signed an informed consent form.

## 3 RESULTS

### 3.1 Addressing the challenges of patient comfort and understanding of the questionnaires and reaching consensus

Thematic analysis of data collected during team meetings identified a six‐step approach to address the above challenges.

#### 3.1.1 Step 1: Recognizing patient partner concerns, discussing to better understand and acknowledge these concerns and reframing the challenges

Patient partners first identified, examined and discussed the challenges regarding the questionnaires amongst themselves and agreed on the main concerns. Specifically, these concerns included the absence of an introduction to the questionnaires, unclear words or phrases, imprecise and/or unnecessary questions, inappropriateness of some questions for participants with multiple conditions and problematic sociodemographic questions, including a lack of consideration for gender diversity. Then, patient partner representatives presented their concerns to the whole PriCARE team. The main difficulty was reconciling the patients' concerns alongside the academic researcher requirements to maintain questionnaire validity. Through discussion and reflection, PriCARE team members recognized patient partners' concerns and determined how to proceed. The team agreed that a prudent first step was to present to the patient partners evidence of the questionnaires' validity and the degree to which patients were engaged in tool development (e.g., whether tool development involved cognitive interviewing with patients or other forms of patient consultation).

#### 3.1.2 Step 2: Detailing and sharing evidence for the validity of the questionnaires

A table, similar to the Supporting Information Appendix S1, detailing the evidence of the instruments' validity and supporting their use was presented to PriCARE team members to support the discussion of potential solutions. For each variable, the PriCARE team members discussed whether other instruments would be more appropriate. Literature searches were undertaken to identify potentially relevant instruments. At the end of this process, based on the criteria for selecting tools, the PriCARE team members concluded that initial instruments were appropriate for the research programme.

#### 3.1.3 Step 3: Evaluating potential solutions

Despite the above‐mentioned concerns raised by the patient partners, the possibility of replacing or introducing significant modifications to the questionnaires was discarded by researchers as this could compromise the psychometric properties of instruments that had already been formally assessed for reliability and validity.^15^ However, it was obvious that the PriCARE team members could not use the questionnaires without introducing minor modifications, such as the inclusion of techniques to promote patient comfort and understanding. Indeed, the ability to establish rapport with patients is one of the most important skills that researchers need to enable engagement and conduct a sensitively presented interview. This rapport can be developed by a range of researcher behaviours, such as paying special attention to the patient, finding common ground, being courteous and empathic, making jokes and sharing personal information when appropriate and providing emotional support. Researcher‐administered questionnaires, rather than self‐administered questionnaires, were encouraged to build this rapport with the patient.^41^ The PriCARE team members also favoured the use of neutral statements and probes that would not affect the validity of the measures (e.g., by saying: *there are no right or wrong answers*), interviewing techniques to encourage response to difficult questions (e.g., by providing clarification to stimulate a response) and reducing the burden of the patient by considering some of the challenging aspects of the questionnaires (to reduce fatigue, consider sensitivity to triggers, distress).^42^

#### 3.1.4 Step 4: Searching the literature for guidelines

The need to adapt questionnaire items for vulnerable and diverse populations has been noted in the literature^15^; yet, few publications discuss addressing this by creating guidelines to facilitate the administration of questionnaires. To develop their own guidelines, the researchers searched the literature for academic publications: (1) providing information to develop guidelines and (2) reporting patients' challenges with the completion of validated questionnaires.

Boynton et al.^16^ propose that representing ‘disempowered and socially excluded groups, cross cultural issues, and participants whose physical and mental health may interfere with their ability to complete a questionnaire’ may be promoted through careful administration of questionnaires in addition to training and support of research staff. The administration of questionnaires is seen as a social interaction that may be challenging for both the participant and the researcher,^13^ and as such, must be rethought to recognize and overcome their inherent bias towards White, well‐educated populations and to adapt them to the needs of larger segments of the population.^13^, ^15^

The World Health Organization regularly develops guidelines for administration to accompany survey instruments.^43^ One such example is the World Health Survey's ‘Guide to administration and question by question specifications’, meant to accompany a survey instrument developed to compile comprehensive baseline information on the health of populations.^42^ The guide is used as a training tool for interviewers when administering the questionnaires and contains instructions, interview guidelines, questionnaire conventions and background information on each question and why it is asked. This manual's structure, the topics that it covered and the intent behind its development have proved useful in the development of similar guidelines for the PriCARE questions. The section covering the use of clarifications, probing techniques and feedback as essential components of standard interviewing techniques was particularly useful.

The PriCARE team members decided to develop guidelines for the administration of the questionnaires that would respond to the challenges identified by patient partners and support researchers in considering the needs of vulnerable patients while introducing only minor modifications to the questionnaires.

#### 3.1.5 Step 5: Creating the guidelines

A working group was formed including one postdoctoral researcher, three research assistants and three patient partners to discuss modifications and create patient‐oriented guidelines for administering the PriCARE questionnaires. Three meetings were held. The first was a brainstorming session to discuss the format and content of the guidelines in response to the challenges posed by the questionnaires and documented in previous meetings of the community of patient partners. In the second meeting, a first draft of the guidelines based on the World Health Survey's ‘Guide to administration and question by question specifications’ was presented and discussed, and modifications to the questionnaires proposed by the patient partners were agreed upon. In the third meeting, a second draft of the guidelines and modified questionnaires was reviewed and prepared for presentation to the larger PriCARE team.

### 3.2 Step 6: Sharing and refining the guidelines

The working group presented the guidelines and questionnaires to the PriCARE team. Team members gave feedback, edited the text and made suggestions to clarify the content of the guidelines. These suggestions were incorporated into the final version of the documents. This version was reviewed and approved by members of the PriCARE team.

### 3.3 Patient‐oriented guidelines for administration of questionnaires

The primary modification to the PriCARE patient questionnaires was the creation of a manual to guide the administration of the PriCARE questionnaires. Researcher‐administered questionnaires are intended to facilitate participant completion, but are considered more labour intensive.^18^ With patient‐oriented guidelines for administration of questionnaires, the responsibility to comprehend and work through challenging questions becomes shared between the participant and the researcher. The manual includes six sections. Section I introduces the PriCARE research programme and the purpose of the questionnaires to ensure that the researcher/interviewer is familiar with the overall research programme. Section II outlines the roles and responsibilities of the researcher and includes training material on interviewing techniques and preparing to administer difficult or sensitive questions. Section III describes how to administer the questionnaires, including how to prepare the respondent and what to say to them before beginning the questionnaires. Section IV reviews the use of neutral probes, feedback and clarifications on an as‐needed basis. Section V includes a copy of the questionnaires with clarifications added beneath questions that patient partners had flagged as challenging. Section VI includes instructions on how to debrief the respondent after completing the questionnaires and end the session. The manual to guide the administration of the PriCARE questionnaires is presented in the Supporting Information Appendix S2.

The second modification to the questionnaires was a format modification^15^ where clarifications were added to challenging questions to reduce respondent burden and enhance comprehension and motivation to complete the questionnaires.^15^ Clarifications are required in situations where the participant is unable to understand the question or the response choices, or expresses difficulty or confusion regarding a questionnaire item.^42^ Short statements were added next to challenging questions, and interviewers were instructed in the guidelines only to use them after neutral probes or feedback failed to stimulate a response.

## 4 DISCUSSION

This is the first study to report the process of researchers and patient partners working together to address challenges of patient comfort and understanding of the questionnaires and to propose patient‐oriented guidelines for administrating the questionnaires. Although the World Health Organization^42^ proposed a guide for administration to accompany survey instruments, their guidelines were developed based on the scientific expertise of academic researchers and did not involve patients.

### 4.1 Process and guidelines adaptable for patient‐oriented projects

With the growing interest worldwide in PROMs,^1^, ^44^, ^45^, ^46^ optimizing patient understanding and comfort during the administration of questionnaires while ensuring validity and respect of psychometric properties becomes an important target to be achieved. Since the balance between comfort and validity remains challenging,^15^, ^16^, ^18^ the six‐step approach to reach consensus with patient partners and the guidelines presented in this paper will be useful for research teams, especially those working with vulnerable populations such as people with chronic conditions, complex needs, cognitive impairments or belonging to minority or migrant groups, highlighting that collaboration.

The six‐step process could be shortened by maintaining Steps 1 and 2 (‘Documenting and discussing the problems’, and ‘Detailing and sharing the evidence for the validity of the questionnaires’) and by adapting the six sections of our guidelines to any research project. Section I of the guidelines could be modified to introduce the new project or programme, the purpose of the questionnaires, with the objective of ensuring the expertise of the researcher/interviewer in the overall research programme. Other sections of the guidelines could be slightly adapted to the new project. Section V should be reviewed to include a copy of the questionnaires with clarifications added beneath questions that patient partners had flagged as challenging. This manual is presented in the Supporting Information Appendix S3.

## 5 CONCLUSIONS

The process of reaching a compromise about questionnaire administration—retaining validated instruments but reviewing their administration—is a powerful demonstration of genuine, collaborative partnership between patients and researchers. This six‐step approach demonstrates how research teams can integrate patient partners into the research team as equal members into the research team and develop meaningful collaboration through recognition of individual experiences and expertise. Ultimately, incorporating these steps ensures that the patient's perspective is expressed through questionnaire research, the development of data collection tools and healthcare innovation in general.

## CONFLICT OF INTERESTS

The authors declare that there are no conflicts of interest.

## AUTHOR CONTRIBUTIONS

Catherine Hudon and Maud‐Christine Chouinard contributed to the PriCARE research programme conception and design. Alya Danish led the different steps for the patient‐oriented guidelines to administration of the questionnaire with the involvement of Dana Howse, Monique Cassidy, Olivier Dumont‐Samson, Judy Porter and Donna Rubenstein. The first draft of the manuscript was written by Catherine Hudon and Alya Danish, and all authors commented on previous versions of the manuscript. All authors read and approved the final manuscript.

## Supporting information

Supplementary Information

Supplementary Information

Supplementary Information

## Data Availability

The data sets supporting the conclusions of this article are included within the article and its additional files.

## References

[1] Weldring T , Smith SM . Patient‐reported outcomes (PROs) and patient‐reported outcome measures (PROMs). Health Serv Insights. 2013;6:61‐68. 10.4137/HSI.S11093 25114561 PMC4089835

[2] U.S. Department of Health HS, FDA, Center for Drug Evaluation Research, U.S. Department of Health Human Services FDA, Center for Biologics Evaluation Research, U.S. Department of Health Human Services FDA, Center for Devices Radiological, Health . Guidance for industry: patient‐reported outcome measures: use in medical product development to support labeling claims: draft guidance. Health Qual Life Outcomes. 2006;4:79. 10.1186/1477-7525-4-79 PMC1629006 17034633

[3] Reeve BB , Wyrwich KW , Wu AW , et al. ISOQOL recommends minimum standards for patient‐reported outcome measures used in patient‐centered outcomes and comparative effectiveness research. Qual Life Res. 2013;22(8):1889‐1905. 10.1007/s11136-012-0344-y 23288613

[4] Wiering B , de Boer D , Delnoij D . Patient involvement in the development of patient‐reported outcome measures: a scoping review. Health Expect. 2017;20(1):11‐23. 10.1111/hex.12442 26889874 PMC5217930

[5] Haywood KL , Staniszewska S , Chapman S . Quality and acceptability of patient‐reported outcome measures used in chronic fatigue syndrome/myalgic encephalomyelitis (CFS/ME): a systematic review. Qual Life Res. 2012;21(1):35‐52. 10.1007/s11136-011-9921-8 21590511

[6] Paterson C . Seeking the patient's perspective: a qualitative assessment of EuroQol, COOP‐WONCA charts and MYMOP. Qual Life Res. 2004;13(5):871‐881. 10.1023/B:QURE.0000025586.51955.78 15233501

[7] Peeters G , Barker AL , Talevski J , et al. Do patients have a say? A narrative review of the development of patient‐reported outcome measures used in elective procedures for coronary revascularisation. Qual Life Res. 2018;27(5):1369‐1380. 10.1007/s11136-018-1795-6 29380228

[8] Wiering B , de Boer D , Delnoij D . Patient involvement in the development of patient‐reported outcome measures: the developers' perspective. BMC Health Serv Res. 2017;17(1):635. 10.1186/s12913-017-2582-8 28886742 PMC5591531

[9] Juniper EF . Validated questionnaires should not be modified. Eur Respir J. 2009;34(5):1015‐1017. 10.1183/09031936.00110209 19880615

[10] Floyd J , Fowler JR , Mangione TW . Standardized Survey Interviewing Minimizing Interviewer‐Related Error. Sage; 1990.

[11] Conrad FG , Schober MF . Clarifying question meaning in a household telephone survey. Public Opin Q. 2000;64(1):1‐28. 10.1086/316757 10810073

[12] Schober MF , Conrad FG , Fricker SS . Misunderstanding standardized language in research interviews. Appl Cogn Psychol. 2004;18:169‐188.

[13] Boynton PM . Administering, analysing, and reporting your questionnaire. BMJ. 2004;328(7452):1372‐1375. 10.1136/bmj.328.7452.1372 15178620 PMC420299

[14] Beadle‐Brown J , Ryan S , Windle K , et al. Engagement of People with Longterm Conditions in Health and Social Care Research: Barriers and Facilitators to Capturing the Views of Seldom‐Heard Populations. Policy Research Unit in Quality and Outcomes of person‐centred care (QORU); 2012.

[15] Stewart AL , Thrasher AD , Goldberg J , Shea JA . A framework for understanding modifications to measures for diverse populations. J Aging Health. 2012;24(6):992‐1017. 10.1177/0898264312440321 22495768 PMC3768261

[16] Boynton PM , Wood GW , Greenhalgh T . Reaching beyond the white middle classes. BMJ. 2004;328(7453):1433‐1436. 10.1136/bmj.328.7453.1433 15191985 PMC421793

[17] De Vries K , Leppa CJ , Sandford R , Vydelingum V . Administering questionnaires to older people: rigid adherence to protocol may deny and disacknowledge emotional expression. J Aging Stud. 2014;31:132‐138. 10.1016/j.jaging.2014.09.005 25456630

[18] Isaksson U , Santamäki‐Fischer R , Nygren B , Lundman B , Åström S . Supporting the very old when completing a questionnaire: risking bias or gaining valid results? Res Aging. 2007;29(6):576‐589. 10.1177/0164027507305924

[19] Danish A , Chouinard MC , Aubrey‐Bassler K , et al. Protocol for a mixed‐method analysis of implementation of case management in primary care for frequent users of healthcare services with chronic diseases and complex care needs. BMJ Open. 2020;10(6):e038241. 10.1136/bmjopen-2020-038241 PMC7265033 32487584

[20] Hudon C , Chouinard MC , Aubrey‐Bassler K , et al. Case management in primary care for frequent users of healthcare services with chronic diseases and complex care needs: an implementation and realist evaluation protocol. BMJ Open. 2018;8(11):e026433. 10.1136/bmjopen-2018-026433 PMC6254422 30478129

[21] Israel BA , Schulz AJ , Parker EA , Becker AB . Review of community‐based research: assessing partnership approaches to improve public health. Annu Rev Public Health. 1998;19:173‐202. 10.1146/annurev.publhealth.19.1.173 9611617

[22] Baum F , MacDougall C , Smith D . Participatory action research. J Epidemiol Community Health. 2006;60(10):854‐857. 10.1136/jech.2004.028662 16973531 PMC2566051

[23] Hudon C , Chouinard M‐CC , Bisson M , et al. Case study with a participatory approach: rethinking pragmatics of stakeholder engagement for implementation research. Ann Fam Med. Accepted. 10.1370/afm.2717 PMC8575520 34750129

[24] Amirav I , Vandall‐Walker V , Rasiah J , Saunders L . Patient and researcher engagement in health research: a parent's perspective. Pediatrics. 2017;140(3):e20164127. 10.1542/peds.2016-4127. 28851740

[25] Chew LD , Griffin JM , Partin MR , et al. Validation of screening questions for limited health literacy in a large VA outpatient population. J Gen Intern Med. 2008;23(5):561‐566. 10.1007/s11606-008-0520-5 18335281 PMC2324160

[26] Hudon É , Hudon C , Couture EM , et al. Measuring health literacy in primary health care: validation of a French‐language version of a three‐item questionnaire. NAPCRG Annual Meeting; 2016; Maryland, USA.

[27] Bayliss EA , Ellis JL , Steiner JF . Subjective assessments of comorbidity correlate with quality of life health outcomes: initial validation of a comorbidity assessment instrument. Health Qual Life Outcomes. 2005;3:51. 10.1186/1477-7525-3-51 PMC1208932 16137329

[28] Poitras M‐E , Fortin M , Hudon C , Haggerty J , Almirall J . Validation of the disease burden morbidity assessment by self‐report in a French‐speaking population. BMC Health Serv Res. 2012;12:35. 10.1186/1472-6963-12-35 PMC3305524 22333434

[29] King J , Gibbons E , Graham C , Walsh J . Developing Measures of People's Self‐Reported Experiences of Integrated Care. Picker Institute Europe & University of Oxford; 2013.

[30] Joober H , Chouinard MC , King J , Lambert M , Hudon E , Hudon C . The patient experience of integrated care scale: a validation study among patients with chronic conditions seen in primary care. Int J Integr Care. 2018;18(4):1. 10.5334/ijic.4163 PMC6199562 30483034

[31] Battersby MW , Ask A , Reece MM , Markwick MJ , Collins JP . The Partners in Health scale: the development and psychometric properties of a generic assessment scale for chronic condition self‐management. Aust J Prim Health. 2003;9(3):41‐52. 10.1071/PY03022

[32] Hudon E , Chouinard MC , Krieg C , et al. The French adaptation and validation of the Partners in Health (PIH) scale among patients with chronic conditions seen in primary care. PLoS One. 2019;14(10):e0224191. 10.1371/journal.pone.0224191 31644561 PMC6808494

[33] Cheak‐Zamora NC , Wyrwich KW , McBride TD . Reliability and validity of the SF‐12v2 in the medical expenditure panel survey. Qual Life Res. 2009;18(6):727‐735. 10.1007/s11136-009-9483-1 19424821

[34] Dauphinee SW , Gauthier L , Gandek B , Magnan L , Pierre U . Readying a US measure of health status, the SF‐36, for use in Canada. Clin Invest Med. 1997;20(4):224‐238. 9258577

[35] Kessler RC , Andrews G , Colpe LJ , et al. Short screening scales to monitor population prevalences and trends in non‐specific psychological distress. Psychol Med. 2002;32:959‐976. 10.1017/s0033291702006074 12214795

[36] Arnaud B , Malet L , Teissedre F , et al. Validity study of Kessler's psychological distress scales conducted among patients admitted to French emergency department for alcohol consumption‐related disorders. Alcohol Clin Exp Res. 2010;34(7):1235‐1245. 10.1111/j.1530-0277.2010.01201.x 20477768

[37] Ishida M , Hulse ES , Mahar RK , et al. The joint effect of physical multimorbidity and mental health conditions among adults in Australia. Prev Chronic Dis. 2020;17:E157. 10.5888/pcd17.200155 33301391 PMC7769083

[38] Krieg C , Hudon C , Chouinard MC , Dufour I . Individual predictors of frequent emergency department use: a scoping review. BMC Health Serv Res. 2016;16(1):594. 10.1186/s12913-016-1852-1 27765045 PMC5072329

[39] Braun V , Clarke V . Using thematic analysis in psychology. Qual Res Psychol. 2006;3:77‐101.

[40] Lincoln YS , Guba EG . Naturalistic Inquiry. Sage; 1985.

[41] Bell K , Fahmy E , Gordon D . Quantitative conversations: the importance of developing rapport in standardised interviewing. Qual Quant. 2016;50:193‐212. 10.1007/s11135-014-0144-2 26792949 PMC4705135

[42] World Health Organization . Guide to Administration and Question by Question Specifications; 2002.

[43] World Health Organization . Assessment of Human Resources for Health. Survey Instruments and Guide to Administration, 2002.

[44] OECD . Recommendations to OECD ministers of health from the high level reflection group on the future of health statistics. Strengthening the international comparison of health system performance through patient‐reported indicators. 2017. Accessed August 25, 2020. https://www.oecd.org/els/health-systems/Recommendations-from-high-level-reflection-group-on-the-future-of-health-statistics.pdf

[45] Greenhalgh J . The applications of PROs in clinical practice: what are they, do they work, and why? Qual Life Res. 2009;18(1):115‐123. 10.1007/s11136-008-9430-6 19105048

[46] Gleeson H , Calderon A , Swami V , Deighton J , Wolpert M , Edbrooke‐Childs J . Systematic review of approaches to using patient experience data for quality improvement in healthcare settings. BMJ Open. 2016;6(8):e011907. 10.1136/bmjopen-2016-011907 PMC5013495 27531733

