# Supplementary material for: Reconciling validity and challenges of patient comfort and understanding: Guidelines to patient‐oriented questionnaires
Source: Health Expect. 2021 Oct 20;25(5):2147–54. doi: 10.1111/hex.13373 (PMC9615088; doi:10.1111/hex.13373)
Supplement: Supplementary file 1 — Supplementary Information [file HEX-25--s001.docx]

**Appendix 1. Description of the instruments used in the PriCARE research program**

| **Variable or**  **outcome** | **Instrument** | **Number of items** | **Psychometrics properties** | | **Cognitive interview*** |
| --- | --- | --- | --- | --- | --- |
|  |  |  | **English-language version** | **French-language version** |  |
| Health literacy | Chew [25-26] | 3 | Effective in detecting inadequate health literacy (area under the receiver operating characteristic curve ranging from 0.76 – 0.87) | Internal consistency: α: 0.77  Test-retest reliability: ICC: 0.69 (95% CI: 0.45 – 0.83) | No |
| Multimorbidity | Disease Burden Morbidity Assessment (DBMA) [27- 28] | 21 | Compared with the chronic disease list in the medical chart as a gold standard:   - Sensitivity: 75% - Specificity: 92% | Compared with the completion of the DBMA based on chart review as gold standard:   - Sensitivity: 74% - Specificity: 92%   Test-retest reliability: ICC: 0.86 (95% CI: 0.79 - 0.92) | Yes |
| Care integration | Patient Experience of Integrated Care Scale [29-30] | 13 |  | Internal consistency: α: 0.88  Test-retest reliability: ICC: 0.81 (95% CI: 0.64 - 0.90)  Concurrent validity with three dimensions of the Continuity of Care from Multiple Clinicians: r_s_: 0.44 - 0.54 | Yes |
| Self-management | Partners in Health Scale [31-32] | 12 | Internal consistency: α: 0.86 | Internal consistency: α: 0.85  Test-retest reliability: ICC: 0.77 (95% CI: 0.58 - 0.87)  Concurrent validity with:   - Self-efficacy for Managing Chronic Disease: r_s_: 0.68 - Patient Activation Measure: r_s_: 0.61 | Yes |
| Quality of life | SF-12v2 [33-34] | 12 | Internal consistency for each subscale:   - PCS: α: 0.88 - MCS: α: 0.82   Test-retest reliability for each subscale:   - PCS: ICC: 0.78 - MCS: ICC: 0.60   Convergent validity for PCS with EQ-5D items (except selfcare) and physical health status: r_s_: 0.56 - 0.61  Convergent validity for MCS with the anxiety/depression item on the EQ-5D: r_s_: 0.61 | Internal consistency for each domain**:   - Physical functioning: α: 0.94 - Role physical: α: 0.88 - Bodily pain: α: 0.94 - General health: α: 0.83 - Vitality: α: 0.80 - Social functioning: α: 0.84 - Role emotional: α: 0.92 - Mental health: α: 0.82 | Yes |
| Psychological distress | Kessler Psychological Distress Scale – 6 items (K6) [35-36] | 6 | Internal consistency: α: 0.89  Effective in detecting mental disorders (area under the receiver operating characteristic curve ranging from 0.88 – 0.96) | Internal consistency: α: 0.76  Effective in detecting depressive symptomatology (area under the receiver operating characteristic curve 0.87) |  |

α : Cronbach’s alpha coefficient; CI: Confidence interval; ICC: intraclass correlation coefficient; MCS : mental component summary; PCS: physical component summary; r_s_: Spearman's rank correlation coefficient.

* It is a qualitative method which consists of conducting interviews with individuals before final validation to analyze how participants understand the questions in a way to ensure the quality and accuracy of the questionnaire.This method is recommended by international guidance on PROM development [2, 3].

**Psychometric proprieties came from a validation study on the SF-36 among a French-Canadian population.
