# Supplementary material for: Reconciling validity and challenges of patient comfort and understanding: Guidelines to patient‐oriented questionnaires
Source: Health Expect. 2021 Oct 20;25(5):2147–54. doi: 10.1111/hex.13373 (PMC9615088; doi:10.1111/hex.13373)
Supplement: Supplementary file 2 — Supplementary Information [file HEX-25--s003.docx]

**
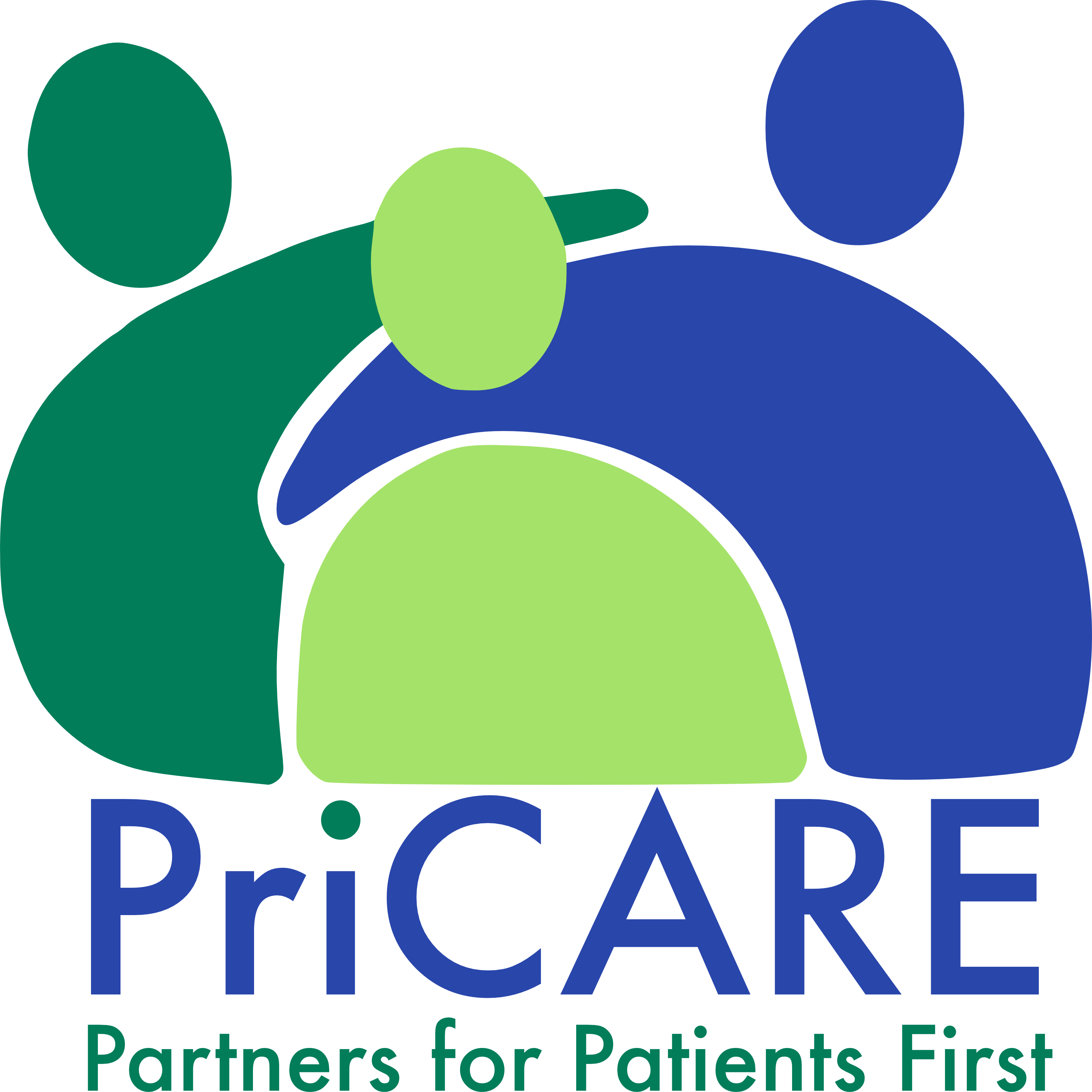
**

**GUIDELINES FOR ADMINISTRATION**

**Patient questionnaires**

*Case management in primary care for frequent users of healthcare services with chronic diseases and complex care needs: implementation and realist evaluations*

Table of Contents

[SECTION I 1](#_Toc67562270)

[GENERAL INFORMATION 1](#_Toc67562271)

[1. BACKGROUND 1](#_Toc67562272)

[2. ABOUT THE QUESTIONNAIRES 1](#_Toc67562273)

[3. PARTICIPANT 2](#_Toc67562274)

[4. ADMINISTERING THE QUESTIONNAIRES 2](#_Toc67562275)

[5. QUESTIONNAIRES CHARACTERISTICS 2](#_Toc67562276)

[SECTION II 4](#_Toc67562277)

[RESPONSIBILITIES OF THE INTERVIEWER 4](#_Toc67562278)

[1. INTERVIEWER INSTRUCTIONS 4](#_Toc67562279)

[2. HOW TO ENCOURAGE RESPONSE TO DIFFICULT QUESTIONS 5](#_Toc67562280)

[3. REVIEWING THE QUESTIONNAIRES 6](#_Toc67562281)

[SECTION III 7](#_Toc67562282)

[HOW TO ADMINISTER THE QUESTIONNAIRES 7](#_Toc67562283)

[1. STUDY BACKGROUND 7](#_Toc67562284)

[2. PARTICIPANTS 7](#_Toc67562285)

[3. OBJECTIVES 7](#_Toc67562286)

[4. TYPES OF QUESTIONS 7](#_Toc67562287)

[5. DURATION OF INTERVIEWS 8](#_Toc67562288)

[6. PRE-INTERVIEW PREPARATION 8](#_Toc67562289)

[7. INSTRUCTIONS TO PARTICIPANT 9](#_Toc67562290)

[SECTION IV 10](#_Toc67562291)

[QUESTION CLARIFICATIONS 10](#_Toc67562292)

[SECTION V 11](#_Toc67562293)

[PATIENT QUESTIONNAIRES 11](#_Toc67562294)

[SECTION VI 12](#_Toc67562295)

[WRAPPING UP 12](#_Toc67562296)

[SECTION VII 13](#_Toc67562297)

[REFERENCES 13](#_Toc67562298)

# SECTION I

## GENERAL INFORMATION

### 1. BACKGROUND

This manual was based on a six-step approach developed by the PriCARE research team to reconcile good research practices for using validated questionnaires and the challenges in questionnaire development related to patient comfort and understanding. The 6-step approach included: 1) Recognizing patient partner concerns, discussing these concerns, and reframing the challenges; 2) Detailing and sharing evidence of the validity of the questionnaires; 3) Evaluating potential solutions; 4) Searching literature for guidelines; 5) Creating guidelines; 6) Sharing and refining guidelines. See Hudon et al., 2021, for more details. It is based on a participatory approach to engage patient partners in research, i.e. to work together with the academic team, to discuss the challenges regarding the questionnaires, review the questionnaires, and come up with different solutions.

This manual is to be used as a training and support tool by research professionals when administering questionnaires. This manual is developed to facilitate the administration of a questionnaire designed to compile comprehensive information about patients enrolled in the research program.

### 2. ABOUT THE QUESTIONNAIRES

The questionnaires used in the PriCARE program will help researchers collect detailed information about the individuals participating in the CM program. The following six questionnaires are included that have been validated in English and in French:

| **Variable or**  **outcome** | **Questionnaire** | **Number of questions** | **Reference** |
| --- | --- | --- | --- |
| Multimorbidity | Disease Burden Morbidity Assessment (DBMA) | 23 | Poitras et al. 2012;  Bayliss et al. 2002 |
| Health literacy | Chew’s questionnaire | 3 | Chew et al. 2004;  Hudon et al. 2016 |
| Care integration | Patient Experience of Integrated Care Scale | 13 | King et al., 2013;  Joober et al., 2018 |
| Self-management | Partners in Health Scale | 12 | Battersby et al., 2003;  Hudon et al., 2019 |
| Quality of life | SF-12v2 | 12 | Cheak-Zamora NC et al., 2009;  Dauphinee SW et al., 1997 |
| Psychological distress | Kessler Psychological Distress Scale – 6 items (K6) | 6 | Kessler et al., 2002 |

In addition, information will be collected regarding the age, gender, marital status, education, occupation, economic status with family income and patient perception of the individual’s economic situation.

### 3. PARTICIPANT

The questionnaires are to be administered to adults who are living with at least one chronic physical or mental illness, are frequent users of healthcare services and who have complex care needs as determined by a health professional.

Individuals who have a prognosis of less than a year or who exhibit a loss of autonomy are ineligible for this research study.

Depending on the participant’s age, maturity and cognition, as well as the place where they come from, some questions or concepts may be more difficult to understand than others.

### 4. ADMINISTERING THE QUESTIONNAIRES

The participant should ideally respond to the questionnaires in a quiet, private place. The questionnaires will be administered by the PriCARE research professional over the phone, or in person if preferred and if possible.

At the point of enrollment in the CM program, the participant may have requested that a family member or caregiver be present for assistance. If this is the case, the family or caregiver must be reminded to refrain from influencing the participant’s answers to the questionnaires.

### 5. QUESTIONNAIRES CHARACTERISTICS

Close-ended questions are used throughout the questionnaires. The participant must choose among the options already provided.

Different types of response choices are used throughout the questionnaires.

In a categorical response choice, the participant will be asked to select a category that best applies to them. For example, the participant will be asked for their relationship status and s/he must state whether s/he are married, living with a partner, separated, divorced, widowed or single.

In a numerical response choice, the participant is asked to provide numbers, such as in a question about date of birth.

In an ordinal response choice, the participant is asked to rate or rank the choices given, such as in a question about whether medical information has been explained by his/her health professional and the participant must choose between “always”, “usually”, “sometimes”, “rarely” or “never”.

# SECTION II

## RESPONSIBILITIES OF THE INTERVIEWER

The interviewer is responsible for becoming familiar with the interview guide, learning the questions and anticipating potential difficulties that may be signalled by the participant.

The interviewer is responsible for asking questions, recording the participant’s answers, addressing the participant’s queries and reviewing the questionnaires before ending the meeting with the participant to ensure that all pertinent information has been recorded.

The interviewer must verify that the participant has understood the questions by providing clarification and appropriate feedback and ensuring that each question has been adequately answered by the participant. The interviewer should take note of questions required clarification and the way s/he addressed this to make sure the messaging is consistent.

The interviewer should set a comfortable pace for the interview and enable the participant to remain focused and interested. By remaining attuned to the participant’s verbal and non-verbal communication, the interviewer can help to ensure a comfortable and pleasant experience for the participant.

### 1. INTERVIEWER INSTRUCTIONS

The interviewer should state their organizational affiliation when introducing him/herself. During in-person interviews, it is recommended that the interviewer wear professional attire and a badge or present their identification (Boynton et al., 2004).

The interviewer must briefly describe the research, the importance of the participant’s contribution and the principles of confidentiality and informed consent.

The interviewer must clearly communicate the objectives of the questionnaires to the participant (please refer to section III.3).

The interviewer should speak clearly and slowly and demonstrate interest in the process.

The interviewer must read the questions as they are written, without changing the wording or the order of the questions, skipping questions or making assumptions regarding the participant’s choices or feedback.

The interview should not be rushed or the participant may feel pressured, resulting in a non-accurate response. Make sure that there is adequate time to address questions and concerns.

The interviewer must remind the participant that the questionnaires are not a test and there are no right or wrong answers.

*The questions in this document may address sensitive or uncomfortable topics. The interviewer should be mindful of observing the participant’s physical or verbal cues for signs of discomfort during the interview.*

*The interviewer may suggest a break and/or offer the participant a glass of water.*

Finally, the interviewer should follow the protocol as outlined in their ethics application.

### 2. HOW TO ENCOURAGE RESPONSE TO DIFFICULT QUESTIONS

According to the World Health Organization (WHO, 2002), providing clarification of a question, probing, or using specific, appropriate feedback is needed when the participant expresses difficulty answering the questions, for example, the participant:

- Is unable to answer the question.
- Does not seem to understand the question.
- Does not seem to have heard the question.
- Hesitates or cannot make up his/her mind.
- Talks about topics or gives responses that are not covered by the questionnaires.
- Needs to expand on what s/he said or clarify their response.
- Asks for a specific part of the question to be repeated. It is acceptable for the interviewer to repeat only that part.
- Asks for one option to be repeated. The interviewer should read all options again but may omit one option if it has clearly been eliminated by the participant.
- Asks for one term to be clarified.

In any of the above situations, it is suggested that the interviewer **first** repeat the question, pause for a brief moment, or repeat the participant’s reply (if applicable) to allow the participant to collect their thoughts and reflect on how they would like to respond (WHO, 2002). At this time, the interviewer should not suggest answers or make assumptions about the participant’s opinion. The interviewer should not appear to be approving or disapproving of the participant or imply that a response is right or wrong.

In addition, the interviewer may use probes which are neutral statements or questions that stimulate response without introducing bias, and feedback, which reassures the participant that they are doing well and can be used to maintain control over the interview to avoid digression. Some examples of probes and feedback are provided in the Table 1 below.

Table 1: Examples of probes and feedback (adapted from WHO, 2002)

| **Probes** | | **Feedback** |
| --- | --- | --- |
| **Statements** | **Questions** |  |
| Overall… | Can you be more specific? | I see.. |
| Generally speaking… | What is your best estimate? | I get your point |
| Whatever … means to you | What do you mean by that? | That is useful information |
| Whatever you think is… | In what sense are you saying that? | It is important to know what your opinion on this is |
| Let me repeat the question again | What do you think | Thank you for your clarification on this |
| Let me repeat the different options again | Which would be closer to your condition? | I understand what you are saying |
| The definition for … is | Would you say that you strongly agree or disagree? | Your comments are very helpful |
| Yes, but… | Can you tell me more about that? | Let me make a note of what you have just said |
| There are no right or wrong answers… | Can you think of any other examples? | Let me make sure I understand correctly |
| We are just interested in your opinion | How is that? In what way? |  |
| Of course, it is difficult to know, but… | Can you explain? |  |
|  | Anything else? |  |

**If** the participant is still expressing difficulty, the interviewer should assist the participant by providing clarification to stimulate response. The interviewer should refer to the clarifications provided underneath each question in the questionnaires, when they exist. These are indicated in italics with a letter *C:* for clarification. Please refer to Section IV for further details.

### 3. REVIEWING THE QUESTIONNAIRES

The interviewer must go through the questionnaires while the participant is still present and make sure that all given responses have been indicated in the appropriate spaces.

The interviewer should also review the coversheet and make sure that required information has been recorded.

# SECTION III

## HOW TO ADMINISTER THE QUESTIONNAIRES

The following sections present the information that should be communicated by the interviewer to the participant before beginning to administer the questionnaires.

### 1. STUDY BACKGROUND

In primary care, people with chronic illnesses have many needs and are confronted with several challenges. In the face of these needs and challenges, project researchers are proposing to set up a follow-up program of CM in primary care to enable people with chronic conditions to receive regular follow-up by a nurse or social worker directly within their primary care clinic.

The aim of this project is to set up a CM program by a nurse or social worker within two (2) primary care clinics in each of the 5 provinces of Newfoundland and Labrador, Nova Scotia, New Brunswick, Quebec and Saskatchewan, for adults with chronic conditions and complex care needs requiring many visits to health and social care services. This program will be evaluated and analyzed by the research team. We hope that the CM program can improve the health of patients, the well-being of health professionals and the health care system (Hudon et al., 2018; Danish et al., 2020).

### 2. PARTICIPANTS

You are among a group of patients from this clinic that has agreed to participate in helping the research team learn more about the CM program.

### 3. OBJECTIVES

The purpose of these questionnaires is to find out more about your individual situation living with a chronic illness in addition to other health or social care needs.

### 4. TYPES OF QUESTIONS

We will ask you questions about many topics, for example:

-your state of health and the illnesses with which you are living,

-your ability to access and use health information to make appropriate decisions and keep yourself healthy (called "health literacy"),

-your perception of the level of integration of the care you receive, your ability to take charge of your health (called “self-management”),

-your quality of life, and sociodemographic information such as your sex, date of birth, place of birth, first language, level of education, occupation, relationship status, and financial income.

We will ask you to participate in this questionnaire on three occasions, at the beginning of the case-management program, 4 and 12 months later.

### 5. DURATION OF INTERVIEWS

The interview is expected to last an average 30 minutes, but may take longer depending on the comprehension and literacy level of the participant.

Participants who have language differences or disabilities; difficulties because of their health condition or their socioeconomic challenges; or are very talkative may take longer to answer questions.

### 6. PRE-INTERVIEW PREPARATION

You may gain personal benefit from your participation in this research project, but we cannot guarantee it.

Furthermore, information gathered from these questionnaires may contribute to the advancement of knowledge in the field of primary care.

You may experience slight fatigue due to the attention required to respond to questionnaires.

Your participation in these questionnaires is voluntary. You are free to refuse to participate.

You can also opt out of this research at any time by informing the research team without having to give reasons.

Your decision not to participate in or to withdraw from this research project will have no effect on the quality of care and services to which you are entitled, or on your relationship with the teams that provide them.

[The interviewer should clearly state the potential risks, disadvantages and advantages of patient participation as outlined in the patient consent form]

### 7. INSTRUCTIONS TO PARTICIPANT

Please feel free to ask me to explain any word or information that is not clear.

If you are not sure how to answer a question, please let me know. I will help you to select the answer that best suits your experience.

If some questions are sensitive or uncomfortable, please let me know if you would like to take a break, get a glass of water, or skip the question.

All your answers will remain confidential.

Do you have any questions?

Do you feel comfortable with what I have just told you?

Do you need anything?

Are you ready to start?

# SECTION IV

## QUESTION CLARIFICATIONS

The patient partners have been invited to review the questionnaires, to express their concerns and to formulate suggestions to promote patient comfort and understanding. In that context, clarifications were added in situations where the participant is unable to understand the question or the response choices, or expresses difficulty or confusion regarding a questionnaire item. This purpose of question clarifications is to provide additional details about each question that may assist the participant in making a response choice.

Interviewers may refer to this information when participants express difficulty with a questionnaire item (See Section II.2 for further details). Interviewers must refrain from offering their own interpretations.

**Question clarifications are coded as *C:***

**Any statement preceded by *C:* should not be read with the original question. The statement should only be used if the participant requests clarification or expresses difficulty answering the question.**

**Probes and feedback can be used at any time to assist the participant.**

# SECTION V

## PATIENT QUESTIONNAIRES

*Items clarifications are presented below for each questionnaire:*

| **Questionnaire** | **Item** | **Clarification** |
| --- | --- | --- |
| Chew’s questionnaire on health literacy | Help read hospital materials | *Such as test results, instructions, prescriptions*. |
|  | Problems learning with written information | *Such as test results, doctors’ messages, remembering conversations or instructions*. |
|  | Confident with forms | *Confident means you are sure of yourself* |
| Patient Experience of Integrated Care Scale | Needs assessed | *Have your needs been identified and understood?* |
|  | Patient involvement in care and support decision | *Involved means to be a part of or associated with.* |
|  | Health care reviewed | *Review means a formal examination or assessment of something, sometimes with the intention of making changes.* |
|  | Information about other services | *Such as meal programs, rehabilitation programs for eating disorders or substance use, and community mental health programs.* |
| Partners in Health Scale | Please circle the number on the scales on the following pages that most closely matches your response for each of the questions below. | *If you have multiple conditions, treatments and health providers, please choose an average (or an in between) score that reflects your overall situation and experience* |
| SF-12v2 | — | *—* |
| Kessler Psychological Distress Scale – 6 items (K6) | — | *—* |
| Disease Burden Morbidity Assessment (DBMA) | Diabetes | *Type 1 or 2* |
|  | Reflux, peptic ulcer or pyrosis | *Heartburn* |
|  | Other, specify | *Such as kidney or blood disorders* |

*.*

# SECTION VI

## WRAPPING UP

Interviewers should allow a few minutes to talk to the participant after the questionnaires have been completed (Boynton et al., 2004).

This will permit a short debriefing with the participant in which the interviewer can repeat the purpose of the questionnaires and the importance of the participant’s participation, and allow the participant to express any thoughts they have regarding the questionnaires or the administration process (Lavrakas, 2008).

The interviewer should solicit and briefly discuss the participant’s questions, comments or concerns.

# SECTION VII

## REFERENCES

Arnaud, B., Malet, L., Teissedre, F., Izaute, M., Moustafa, F., Geneste, J., ... & Brousse, G. Validity study of Kessler’s psychological distress scales conducted among patients admitted to French emergency department for alcohol consumption–related disorders. Alcoholism: Clinical and Experimental Research, 2010, 34(7), 1235-1245.

Bayliss EA, Ellis JL, Steiner JF. Subjective assessments of comorbidity correlate with quality of life health outcomes: initial validation of a comorbidity assessment instrument. Health Qual Life Outcomes, 2005;3:51.

Battersby MW, Ask A, Reece MM, Markwick MJ, Collins JP. The Partners in Health scale: The development and psychometric properties of a generic assessment scale for chronic condition self-management. Australian Journal of Primary Health. 2003,9:41-52.

Boynton PM, Wood GW, Greenhalgh T. Hands-on guide to questionnaire research: Reaching beyond the white middle class. BMJ, 2004; 328: 1433-6.

Brazier JE, Roberts J. The estimation of a preference-based measure of health from the SF-12. Med Care, 2004;42:851–9.

Chew LD, Bradley KA, Boyko EJ. Brief questions to identify patients with inadequate health literacy. Fam Med, 2004;36:588–94.

Curtis E.A. and Drennan J. (eds.) Quantitative Health Research: Issues and methods. Maidenhead: McGraw-Hill Education, 2013

Danish A, Chouinard M-C, Aubrey-Bassler K, et al.Protocol for a mixed-method analysis of implementation of case management in primary care for frequent users of healthcare services with chronic diseases and complex care needs. BMJ Open, 2020;10:e038241. doi:10.1136/bmjopen-2020-038241.

Hudon C, Chouinard M-C, Aubrey-Bassler K, et al. Case management in primary care for frequent users of healthcare services with chronic diseases and complex care needs: an implementation and realist evaluation protocol. BMJ Open, 2018;8:e026433. doi:10.1136/bmjopen-2018-026433.

Hudon E, Hudon C, Couture EM, et al. Measuring health literacy in primary health care: validation of a French-Language version of a Three-Item questionnaire. Colorado Springs, USA: North American Primary Care Research Group Annual Meeting, 2016a.

Hudon E, Hudon C, Lambert M, et al. Validation of a French-language version of a patient-reported measure of integrated care. Colorado Springs, USA: North American Primary Care Research Group Annual Meeting, 2016b.

Hudon E, Chouinard MC, Krieg C, Lambert M, Joober H, Lawn S, et al. The French adaptation and validation of the Partners in Health (PIH) scale among patients with chronic conditions seen in primary care. PLoS One. 2019,14:e0224191.

Hudon C, Danish A, Lambert M, Howse D, et al. Reconciling validity and challenges of patient comfort and understanding: Guidelines to patient-oriented questionnaires, 2021**.**

Joober H, Chouinard MC, King J, Lambert M, Hudon E, Hudon C. The Patient Experience of Integrated Care Scale: A Validation Study among Patients with Chronic Conditions Seen in Primary Care. Int J Integr Care. 2018,18:1.

Kessler RC, Andrews G, Colpe LJ, Hiripi E, Mroczek DK, Normand S-LT, Walters EE, Zaslavsky AM. Short screening scales to monitor population prevalences and trends in non-specific psychological distress. Psychol Med, 2002, 32:959–76.

King J, Gibbons E, Graham C, Walsh J. Developing measures of people’s self-reported experiences of integrated care. Oxford: Picker Institute Europe & University of Oxford; 2013.

Lavrakas P. Encyclopedia of Survey Research Methods. Thousand Oaks: SAGE Publications, 2008.

Poitras M-E, Fortin M, Hudon C, et al. Validation of the disease burden morbidity assessment by self-report in a French-speaking population. BMC Health Serv Res, 2012;12:35.

Smith D, Harvey P, Lawn S, et al. Measuring chronic condition self-management in an Australian community: factor structure of the revised partners in health (PIH) scale. Qual Life Res, 2017;26:149–59.

Walters EE, Zaslavsky AM (2002) Short screening scales to monitor population prevalences and trends in non-specific psychological distress. PsycholMed 32:959–76.

Ware J. User Manuel for the SF-36v2 health survey second edition. Lincoln: Quality Metric Incorporated, 2007.

World Health Survey: Guide to administration and question by question specifications. World Health Organization, 2002.
