# Supplementary material for: Reconciling validity and challenges of patient comfort and understanding: Guidelines to patient‐oriented questionnaires
Source: Health Expect. 2021 Oct 20;25(5):2147–54. doi: 10.1111/hex.13373 (PMC9615088; doi:10.1111/hex.13373)
Supplement: Supplementary file 3 — Supplementary Information [file HEX-25--s002.docx]

**
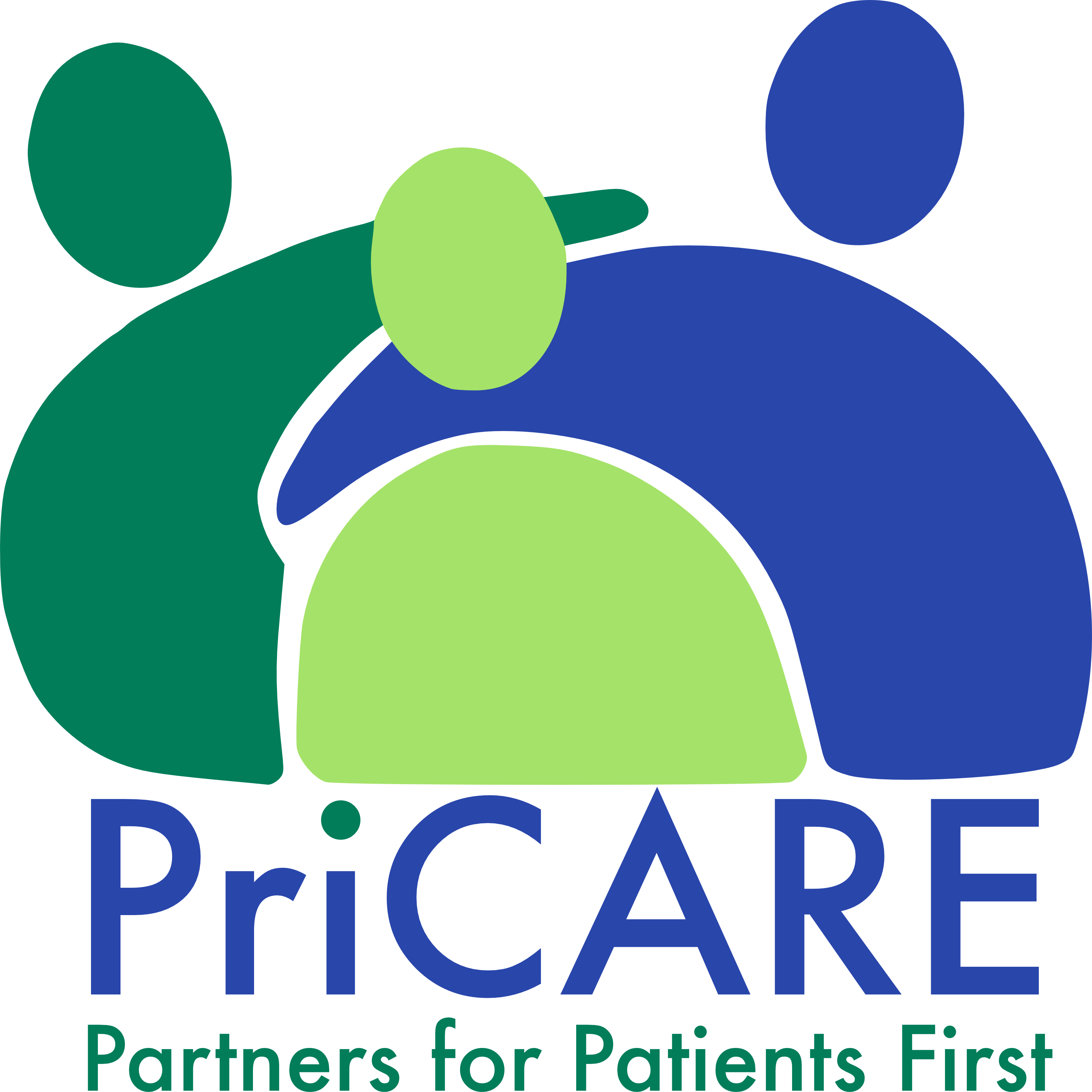
**

**GUIDELINES FOR ADMINISTRATION**

**Patient questionnaires**

Table of Contents

[SECTION I 1](#_Toc67562494)

[GENERAL INFORMATION 1](#_Toc67562495)

[1. BACKGROUND 1](#_Toc67562496)

[2. ABOUT THE QUESTIONNAIRES 1](#_Toc67562497)

[3. PARTICIPANT 2](#_Toc67562498)

[4. ADMINISTERING THE QUESTIONNAIRES 2](#_Toc67562499)

[5. QUESTIONNAIRES CHARACTERISTICS 2](#_Toc67562500)

[SECTION II 3](#_Toc67562501)

[RESPONSIBILITIES OF THE INTERVIEWER 3](#_Toc67562502)

[1. INTERVIEWER INSTRUCTIONS 3](#_Toc67562503)

[2. HOW TO ENCOURAGE RESPONSE TO DIFFICULT QUESTIONS 4](#_Toc67562504)

[3. REVIEWING THE QUESTIONNAIRES 6](#_Toc67562505)

[SECTION III 6](#_Toc67562506)

[HOW TO ADMINISTER THE QUESTIONNAIRES 6](#_Toc67562507)

[1. STUDY BACKGROUND 6](#_Toc67562508)

[2. PARTICIPANTS 6](#_Toc67562509)

[3. OBJECTIVES 6](#_Toc67562510)

[4. TYPES OF QUESTIONS 6](#_Toc67562511)

[5. DURATION OF INTERVIEWS 7](#_Toc67562512)

[6. PRE-INTERVIEW PREPARATION 7](#_Toc67562513)

[7. INSTRUCTIONS TO PARTICIPANT 7](#_Toc67562514)

[SECTION IV 8](#_Toc67562515)

[QUESTION CLARIFICATIONS 8](#_Toc67562516)

[SECTION V 9](#_Toc67562517)

[PATIENT QUESTIONNAIRES 9](#_Toc67562518)

[SECTION VI 9](#_Toc67562519)

[WRAPPING UP 9](#_Toc67562520)

[SECTION VII 10](#_Toc67562521)

[REFERENCES 10](#_Toc67562522)

# SECTION I

## GENERAL INFORMATION

### 1. BACKGROUND

This manual was based on a six-step approach developed by the PriCARE research team to reconcile good research practices for using validated questionnaires and the challenges in questionnaire development related to patient comfort and understanding. The 6-step approach included: 1) Recognizing patient partner concerns, discussing these concerns, and reframing the challenges; 2) Detailing and sharing evidence of the validity of the questionnaires; 3) Evaluating potential solutions; 4) Searching literature for guidelines; 5) Creating guidelines; 6) Sharing and refining guidelines. See Hudon et al., 2021, for more details. It is based on a participatory approach to engage patient partners in research, i.e. to work together with the academic team, to discuss the challenges regarding the questionnaires, review the questionnaires, and come up with different solutions.

This manual is to be used as a training and support tool by research professionals when administering questionnaires. This manual is developed to facilitate the administration of a questionnaire designed to compile comprehensive information about patients enrolled in the research program.

### 2. ABOUT THE QUESTIONNAIRES

The questionnaires used in this study will help researchers collect detailed information about the participants. The following questionnaires are included:

| **Variable or**  **outcome** | **Questionnaire** | **Number of questions** | **Reference** |
| --- | --- | --- | --- |
|  |  |  |  |
|  |  |  |  |
|  |  |  |  |
|  |  |  |  |
|  |  |  |  |
|  |  |  |  |

In addition, information will be collected regarding [sociodemographic variables collected in the study].

### 3. PARTICIPANT

The questionnaires are to be administered to individuals who [study inclusion criteria].

Individuals who [study exclusion criteria] are ineligible for this research study.

Depending on the participant’s age, maturity and cognition, as well as the place where they come from, some questions or concepts may be more difficult to understand than others.

Participants who have language differences or disabilities; difficulties because of their health condition or their socioeconomic challenges; or are very talkative may take longer to answer questions.

### 4. ADMINISTERING THE QUESTIONNAIRES

The participant should ideally respond to the questionnaires in a quiet, private place. The questionnaires will be administered by the research professional [mode of administration].

The participant may have requested that a family member or caregiver be present for assistance. If this is the case, the family or caregiver must be reminded to refrain from influencing the participant’s answers to the questionnaires.

### 5. QUESTIONNAIRES CHARACTERISTICS

Close-ended questions are used throughout the questionnaires. The participant must choose among the options already provided.

Different types of response choices are used throughout the questionnaires.

In a categorical response choice, the participant will be asked to select a category that best applies to them. For example, the participant will be asked for their relationship status and s/he must state whether s/he are married, living with a partner, separated, divorced, widowed or single.

In a numerical response choice, the participant is asked to provide numbers, such as in a question about date of birth.

In an ordinal response choice, the participant is asked to rate or rank the choices given, such as in a question about whether medical information has been explained by his/her health professional and the participant must choose between “always”, “usually”, “sometimes”, “rarely” or “never”.

# SECTION II

## RESPONSIBILITIES OF THE INTERVIEWER

The interviewer is responsible for becoming familiar with the interview guide, learning the questions and anticipating potential difficulties that may be signalled by the participant.

The interviewer is responsible for asking questions, recording the participant’s answers, addressing the participant’s queries and reviewing the questionnaires before ending the meeting with the participant to ensure that all pertinent information has been recorded.

The interviewer must verify that the participant has understood the questions by providing clarification and appropriate feedback and ensuring that each question has been adequately answered by the participant. The interviewer should take note of questions required clarification and the way s/he addressed this to make sure the messaging is consistent.

The interviewer should set a comfortable pace for the interview and enable the participant to remain focused and interested. By remaining attuned to the participant’s verbal and non-verbal communication, the interviewer can help to ensure a comfortable and pleasant experience for the participant.

### 1. INTERVIEWER INSTRUCTIONS

The interviewer should state their organizational affiliation when introducing him/herself. During in-person interviews, it is recommended that the interviewer wear professional attire and a badge or present their identification (Boynton et al., 2004).

The interviewer must briefly describe the research, the importance of the participant’s contribution and the principles of confidentiality and informed consent.

The interviewer must clearly communicate the objectives of the questionnaires to the participant (please refer to section III.3).

The interviewer should speak clearly and slowly and demonstrate interest in the process.

The interviewer must read the questions as they are written, without changing the wording or the order of the questions, skipping questions or making assumptions regarding the participant’s choices or feedback.

The interview should not be rushed or the participant may feel pressured, resulting in a non-accurate response. Make sure that there is adequate time to address questions and concerns.

The interviewer must remind the participant that the questionnaires are not a test and there are no right or wrong answers.

*The questions in this document may address sensitive or uncomfortable topics. The interviewer should be mindful of observing the participant’s physical or verbal cues for signs of discomfort during the interview.*

*The interviewer may suggest a break and/or offer the participant a glass of water.*

Finally, the interviewer should follow the protocol as outlined in their ethics application.

### 2. HOW TO ENCOURAGE RESPONSE TO DIFFICULT QUESTIONS

According to the World Health Organization (WHO, 2002), providing clarification of a question, probing, or using specific, appropriate feedback is needed when the participant expresses difficulty answering the questions, for example, the participant:

- Is unable to answer the question.
- Does not seem to understand the question.
- Does not seem to have heard the question.
- Hesitates or cannot make up his/her mind.
- Talks about topics or gives responses that are not covered by the questionnaires.
- Needs to expand on what s/he said or clarify their response.
- Asks for a specific part of the question to be repeated. It is acceptable for the interviewer to repeat only that part.
- Asks for one option to be repeated. The interviewer should read all options again but may omit one option if it has clearly been eliminated by the participant.
- Asks for one term to be clarified.

In any of the above situations, it is suggested that the interviewer **first** repeat the question, pause for a brief moment, or repeat the participant’s reply (if applicable) to allow the participant to collect their thoughts and reflect on how they would like to respond (WHO, 2002). At this time, the interviewer should not suggest answers or make assumptions about the participant’s opinion. The interviewer should not appear to be approving or disapproving of the participant or imply that a response is right or wrong.

In addition, the interviewer may use probes which are neutral statements or questions that stimulate response without introducing bias, and feedback, which reassures the participant that they are doing well and can be used to maintain control over the interview to avoid digression. Some examples of probes and feedback are provided in the Table 1 below.

Table 1: Examples of probes and feedback (adapted from WHO, 2002)

| **Probes** | | **Feedback** |
| --- | --- | --- |
| **Statements** | **Questions** |  |
| Overall… | Can you be more specific? | I see.. |
| Generally speaking… | What is your best estimate? | I get your point |
| Whatever … means to you | What do you mean by that? | That is useful information |
| Whatever you think is… | In what sense are you saying that? | It is important to know what your opinion on this is |
| Let me repeat the question again | What do you think | Thank you for your clarification on this |
| Let me repeat the different options again | Which would be closer to your condition? | I understand what you are saying |
| The definition for … is | Would you say that you strongly agree or disagree? | Your comments are very helpful |
| Yes, but… | Can you tell me more about that? | Let me make a note of what you have just said |
| There are no right or wrong answers… | Can you think of any other examples? | Let me make sure I understand correctly |
| We are just interested in your opinion | How is that? In what way? |  |
| Of course, it is difficult to know, but… | Can you explain? |  |
|  | Anything else? |  |

**If** the participant is still expressing difficulty, the interviewer should assist the participant by providing clarification to stimulate response. The interviewer should refer to the clarifications provided underneath each question in the questionnaires, when they exist. These are indicated in italics with a letter *C:* for clarification. Please refer to Section IV for further details.

### 3. REVIEWING THE QUESTIONNAIRES

The interviewer must go through the questionnaires while the participant is still present and make sure that all given responses have been indicated in the appropriate spaces.

The interviewer should also review the coversheet and make sure that required information has been recorded.

# SECTION III

## HOW TO ADMINISTER THE QUESTIONNAIRES

The following sections present the information that should be communicated by the interviewer to the participant before beginning to administer the questionnaires.

### 1. STUDY BACKGROUND

[Background and objectives of the study].

### 2. PARTICIPANTS

You are among a group of people that has agreed to participate in helping the research team learn more about [primary focus of the study].

### 3. OBJECTIVES

The purpose of these questionnaires is to find out more about [main subject of the questionnaires].

### 4. TYPES OF QUESTIONS

We will ask you questions about many topics, for example [mains topics of the questionnaires]:

We will ask you to participate in this questionnaires on [number of time points] occasions, [time points].

### 5. DURATION OF INTERVIEWS

The interview is expected to last an average [estimated time to complete the questionnaires] but may take longer depending on the comprehension and literacy level of the participant.

Participants who have language differences or disabilities; difficulties because of their health condition or their socioeconomic challenges; or are very talkative may take longer to answer questions.

### 6. PRE-INTERVIEW PREPARATION

[The interviewer should clearly state the potential risks, disadvantages and advantages of patient participation as outlined in the patient consent form]

### 7. INSTRUCTIONS TO PARTICIPANT

Please feel free to ask me to explain any word or information that is not clear.

If you are not sure how to answer a question, please let me know. I will help you to select the answer that best suits your experience.

If some questions are sensitive or uncomfortable, please let me know if you would like to take a break, get a glass of water, or skip the question.

All your answers will remain confidential.

Do you have any questions?

Do you feel comfortable with what I have just told you?

Do you need anything?

Are you ready to start?

# SECTION IV

## QUESTION CLARIFICATIONS

It is suggested that the research team works with patient partners to review the questionnaires, to express their concerns and to formulate suggestions to promote patient comfort and understanding. In that context, clarifications could be added in situations where the participant is unable to understand the question or the response choices, or expresses difficulty or confusion regarding a questionnaire item. This purpose of question clarifications is to provide additional details about each question that may assist the participant in making a response choice.

Interviewers may refer to this information when participants express difficulty with a questionnaire item (See Section II.2 for further details). Interviewers must refrain from offering their own interpretations.

**Question clarifications are coded as *C:***

**Any statement preceded by *C:* should not be read with the original question. The statement should only be used if the participant requests clarification or expresses difficulty answering the question.**

**Probes and feedback can be used at any time to assist the participant.**

# SECTION V

## PATIENT QUESTIONNAIRES

[Patient questionnaires, including question clarifications]

# SECTION VI

## WRAPPING UP

Interviewers should allow a few minutes to talk to the participant after the questionnaires have been completed (Boynton et al., 2004).

This will permit a short debriefing with the participant in which the interviewer can repeat the purpose of the questionnaires and the importance of the participant’s participation, and allow the participant to express any thoughts they have regarding the questionnaires or the administration process (Lavrakas, 2008).

The interviewer should solicit and briefly discuss the participant’s questions, comments or concerns.

# SECTION VII

## REFERENCES

Boynton PM, Wood GW, Greenhalgh T. Hands-on guide to questionnaire research: Reaching beyond the white middle class. BMJ 2004; 328: 1433-6.

Curtis E.A. and Drennan J. (eds.) Quantitative Health Research: Issues and methods. Maidenhead: McGraw-Hill Education, 2013.

Hudon C, Danish A, Lambert M, Howse D, et al. Reconciling validity and challenges of patient comfort and understanding: Guidelines to patient-oriented questionnaires, 2021**.**

Lavrakas P. Encyclopedia of Survey Research Methods. Thousand Oaks : SAGE Publications, 2008.

World Health Survey: Guide to administration and question by question specifications. World Health Organization, 2002.
